# Assessment of the appropriate cutoff points for anthropometric indices and their relationship with cardio-metabolic indices to predict the risk of metabolic associated fatty liver disease

**DOI:** 10.1186/s12902-024-01615-3

**Published:** 2024-06-04

**Authors:** Seyed Ahmad Hosseini, Meysam Alipour, Sara Sarvandian, Neda Haghighat, Hadi Bazyar, Ladan Aghakhani

**Affiliations:** 1 Nutrition and Metabolic Disease Research CenterClinical Sciences Research Institute, Ahvaz Jundishapur University of Medical Sciences, Ahvaz, Iran; 2 Department of Nutrition, Shoushtar Faculty of Medical Sciences, Shoushtar, Iran; 3 Laparoscopy Research Center, Shiraz University of Medical Sciences, Shiraz, Iran; 4 Department of Public Health, Sirjan School of Medical Sciences, Sirjan, Iran; 5 Student Research Committee, Sirjan School of Medical Sciences, Sirjan, Iran

**Keywords:** Nutrition assessment, Fatty liver, Metabolic diseases, Cardiovascular diseases, Body mass index

## Abstract

**Background:**

Research on Metabolic Associated Fatty Liver Disease (MAFLD) is still in its early stages, with few studies available to identify and predict effective indicators of this disease. On the other hand, early diagnosis and intervention are crucial to reduce the burden of MAFLD. Therefore, the aim of this research was to investigate the effectiveness of eleven anthropometric indices and their appropriate cut-off values as a non-invasive method to predict and diagnose MAFLD in the Iranian population.

**Methods:**

In this cross-sectional study, we analyzed baseline data from the Hoveyzeh Cohort Study, a prospective population-based study conducted in Iran that enrolled a total of 7836 subjects aged 35 to 70 years from May 2016 through August 2018.

**Results:**

The optimal cut-off values of anthropometric indices for predicting MAFLD risk were determined for waist circumference(WC) (102.25 cm for males and 101.45 cm for females), body mass index (BMI) (27.80 kg/m^2^ for males and 28.75 kg/m^2^ for females), waist-to-hip ratio (WHR) (0.96 for both males and females), waist-to-height ratio (WHtR) (0.56 for males and 0.63 for females), body adiposity index (BAI) (23.24 for males and 32.97 for females), visceral adiposity index (VAI) (1.64 for males and 1.88 for females), weight-adjusted waist index (WWI) (10.63 for males and 11.71 for females), conicity index (CI) (1.29 for males and 1.36 for females), body roundness index (BRI) (4.52 for males and 6.45 for females), relative fat mass (RFM) (28.18 for males and 44.91 for females) and abdominal volume index (AVI) (18.85 for males and for 21.37 females). VAI in males (sensitivity: 77%, specificity: 60%, Youden’s Index: 0.37) and RFM in females (sensitivity: 76%, specificity: 59%, Youden’s Index: 0.35) were found to have higher sensitivity and specificity compared to other anthropometric indices. Furthermore, anthropometric indices demonstrated statistically significant correlations with various hepatic and cardiometabolic indices. Among these, the strongest positive correlations were observed between WC, BMI, BAI, BRI, and AVI with the Hepatic Steatosis Index (HSI), TyG-BMI, and TyG-WC, as well as between VAI and the Atherogenic Index of Plasma (AIP), Lipid Accumulation Product (LAP), Cardiometabolic Index (CMI), and the Triglyceride and Glucose (TyG) Index.

**Conclusion:**

Anthropometric indices are effective in predicting MAFLD risk among Iranian adults, with WWI, VAI, and RFM identified as the strongest predictors. The proposed cutoff values could serve as a straightforward and non-invasive methods for the early diagnosis of MAFLD.

## Introduction

A group of international experts from 22 countries proposed a new concept for fatty liver disease in 2020, which is called metabolic (dysfunction) associated with fatty liver disease (MAFLD) [1]. As a result, the condition previously known as non-alcoholic fatty liver disease (NAFLD) has been renamed to MAFLD, which enables the identification of a larger number of individuals with fatty liver [2].

Additionally, the transition from NAFLD to MAFLD has been endorsed by several organizations, including the European Liver Patients’ Association [3]. MAFLD is defined as a condition characterized by hepatic steatosis, as well as the presence of at least one metabolic dysfunction, including overweight or obesity (body mass index ≥ 25 kg/m2), arterial hypertension, diabetes, cardiovascular diseases (CVD), and dyslipidemia [4–6].

Approximately 25% of the global adult population is affected by MAFLD, imposing significant burdens on individuals, economies, and healthcare systems [6, 7]. The underlying mechanisms and processes that contribute to the development and progression of MAFLD are still not well understood. Genetic variation, type 2 diabetes mellitus (T2DM), insulin resistance (IR), dyslipidemia, and cardiovascular disease (CVD) are all examples of associative factors [8].

Furthermore, the increasing prevalence of MAFLD is linked to the rising prevalence of obesity [9]. According to a recent systematic review and meta-analysis, it was found that MAFLD prevalence among overweight or obese adults in the general population was 50.7% [10]. Several studies have also found strong associations between MAFLD and anthropometric measurements, such as body mass index (BMI), waist circumference (WC), and waist-to-height ratio (WHtR) [11, 12].

Obesity has a significant impact on the liver due to the presence of adipokines, which are hormones produced by adipose tissue. Adipokines play critical roles in the development of various stages of MAFLD, including steatosis, nonalcoholic steatohepatitis (NASH), cirrhosis, and carcinogenesis [13].

The high prevalence of cardiometabolic diseases worldwide, which are closely linked to obesity, makes cardiometabolic health a significant public health issue [14, 15]. In addition, there is ample evidence of clear associations between NAFLD and various cardiometabolic disorders, such as ischemic stroke, insulin resistance, hypertension, cardiac arrhythmias, and dyslipidemia [16, 17]. A study showed that MAFLD criteria are more effective than those for NAFLD in identifying individuals at elevated risk for cardiovascular disease [1]. Furthermore, based on the study by Kim et al. [18], MAFLD is associated with a higher risk of cardiovascular mortality.

Although MAFLD is highly prevalent worldwide, there are currently no approved treatments available for this condition [12]. Early diagnosis and assessment of the associated risk factors would greatly aid in the prevention or management of MAFLD. Therefore, this study was conducted to assess the predictive ability of anthropometric indices and determine their optimal cutoff values for predicting MAFLD risk in the Iranian population.

## Materials and methods

### Patients and study design

**Fig. 1:**
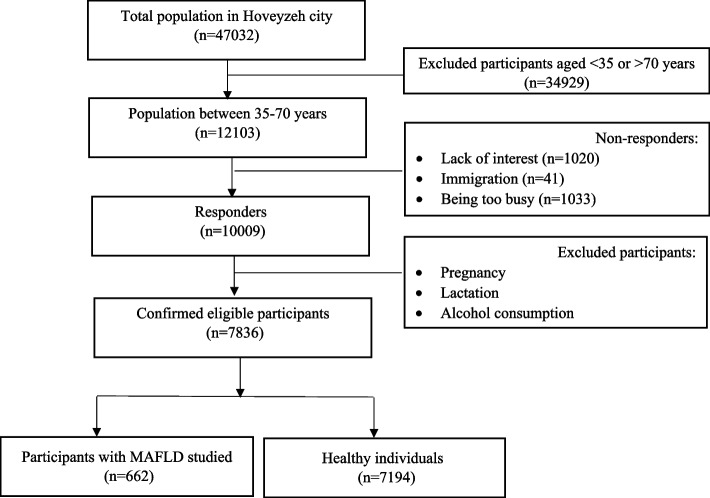
Flow diagram of study selection

The cross-sectional study was part of the Hoveyzeh Cohort Study, a prospective population-based study of noncommunicable diseases in an Arab community in southwestern Iran. This study targeted adults aged 35 to 70 years, from May 2016 through August 2018 [19]. According to Fig. 1, among the respondents (*n* = 10,009) in the city of Hoveyzeh, 7836 individuals were evaluated using inclusion and exclusion criteria. Inclusion criteria included willingness to participate in the study and being between 35 and 70 years old. Exclusion criteria included pregnancy, lactation, and alcohol consumption.Finally, after excluding subjects with incomplete or illogical data related to anthropometric and cardiometabolic indices, 7836 participants, comprising 3190 males and 4646 females, were eligible for the study. Out of these, 642 were diagnosed with MAFLD as NAFLD patients with at least one of the following three conditions: obesity, type 2 diabetes mellitus, and metabolic dysregulation.

### Data collection

All data related to this study were recorded in the Hoveyzeh Cohort Study database and extracted for this research.

#### Anthropometric indices

The anthropometric assessment included measurements of weight, height, WC, wrist, hip circumference (HC), BMI, WHR, WHtR, BAI, VAI, WWI, CI, BRI, RFM, and AVI were calculated.

Body weight (in kg) was measured using a standing scale (Seca 755), and height (in cm) was measured with a stadiometer (Seca 206). WC, wrist circumference (in cm), and HC were measured using Seca locked tape meters. All measurements were performed by trained nursing staff using standard protocols and techniques.Anthropometric indicators were calculated using the following standard equations:

$$\mathrm{BMI}\hspace{0.17em}=\hspace{0.17em}\mathrm{weight}\left(\mathrm{kg}\right)/\mathrm{height}\left(\mathrm m\right)^2$$

$$\mathrm{WHR}\hspace{0.17em}=\hspace{0.17em}\mathrm{WC}\left(\mathrm{cm}\right)/\mathrm{HC}\left(\mathrm{cm}\right)$$

$$\mathrm{WHtR}\hspace{0.17em}=\hspace{0.17em}\mathrm{WC}\left(\mathrm{cm}\right)/\mathrm{height}\left(\mathrm{cm}\right)$$

$$\mathrm{BAI}=\lbrack\mathrm{HC}(\mathrm{cm})/\mathrm{height}(\mathrm m)^{1.5}\rbrack-18$$

$$\mathrm{ABSI}=\mathrm{WC (m)} / \mathrm{[{BMI}^{2/3}(kg/m^{2}) height^{1/2} (m)]}$$

$$\mathrm{VAI}\,=\,(\mathrm{WC}\,(\mathrm{cm})\,/\,\lbrack39.68\,+\,(1.88\,\times\,\mathrm{BMI})\rbrack\,\times\,(\mathrm{TG}\,(\mathrm{mmol}/\mathrm L)/1.03)\,\times\,(1.31/\,\mathrm{HDL}\,(\mathrm{mmol}/\mathrm L))\,\mathrm{in}\,\mathrm{males}$$

$$\mathrm{VAI}\,=\,(\mathrm{WC}\,(\mathrm{cm})\,/\,\lbrack36.58\,+\,(1.89\,\times\,\mathrm{BMI})\rbrack\,\times\,(\mathrm{TG}\,(\mathrm{mmol}/\mathrm L)/0.81)\,\times\,(1.52/\,\mathrm{HDL}\,(\mathrm{mmol}/\mathrm L))\,\mathrm{in}\,\mathrm{females}$$

$$\mathrm{WWI}\hspace{0.17em}=\hspace{0.17em}\mathrm{WC}\left(\mathrm{cm}\right)/\mathrm{Weight}\left(\mathrm{kg}\right)^2$$

$$\mathrm{CI}\hspace{0.17em}=\hspace{0.17em}\mathrm{WC}\left(\mathrm m\right)/\lbrack0.109\times\sqrt{\mathrm{weight}\left(\mathrm{kg}\right)/\mathrm{Height}\left(\mathrm m\right)}\rbrack$$

$$BRI=364.2-365.5\times(1-[\mathrm{WC}/2\pi]^2/[0.5\times\mathrm{height}^2)^{\frac{1}{2}}$$

$$RFM\hspace{0.17em}=\hspace{0.17em}64 - (20 \times height/WC (cm\left)\right) + (12 \times sex); sex\hspace{0.17em}=\hspace{0.17em}0\, for\, males\, and\, 1\,for\,females$$

$$\mathrm{AVI}=\lbrack2\times\mathrm{WC}\left(\mathrm{cm}\right)^2+0.7\times(\mathrm{WC}\left(\mathrm{cm}\right)-\mathrm{HC}{\left(\mathrm{cm}\right))}^2\rbrack/1000$$

#### Biochemical test and blood pressure measurement

Venous blood (10 ml) was taken from all patients after a 12-hour overnight fasting period.The blood samples were centrifuged, and the serums were stored at -70 ° C. A complete blood count (CBC) was performed using the hematology autoanalyser (Nihon Kohden 6510-k, Japan). Serum glucose, triglyceride (TG), total cholesterol (TC), and high-density lipoprotein cholesterol (HDL-C) levels were assessed using a commercial kit (Pars Azmoon, Tehran, Iran). Serum low-density lipoprotein cholesterol (LDL) was calculated using the Friedewald equation.

Brachial blood pressure (BP) was measured in the supine position after at least 10 min of rest. Triple brachial blood pressure recording was performed on the right arm using a validated automated oscillometric device.

Mean Arterial Pressure $\left(MAP\right) = [Systolic BP + (2 \times Diastolic BP\left)\right]/3$  

#### Cardiometabolic and hepatic indices

Cardiometabolic indices were calculated using the following formulas:

The atherogenic index of plasma $\left(AIP\right)\hspace{0.17em}=\hspace{0.17em}Log (TG/ HDL-C)$

lipid accumulation product $\left(LAP\right) = \left[WC \right(cm)-65] \times \left[TG\right] in males, \left[WC \right(cm)-58] \times \left[TG\right]$ in females

Lipoprotein combine index $\left(LCI\right)\hspace{0.17em}=\hspace{0.17em}TC\times TG\times LDL/HDL-C$

Cardiometabolic index $$\left(CMI\right)\hspace{0.17em}=\hspace{0.17em}TG/HDL-C \times WHtR$$

$$TyG\ index: Ln \left[TG \right(mg/dl) \times FPG (mg/dl)/2]$$

$$TyG-BMI: TyG index \times BMI$$

$$TyG-WC: TyG index \times waist circumference \left(cm\right)$$

Thrombolysis in Myocardial Infarction $\left(TIMI risk index\right) score\hspace{0.17em}=\hspace{0.17em}heart rate \left(bpm\right) \times (age/{10)}^{2} /systolic BP (mmHg)$

Cardiovascular risk index $\left(CRI\right)\hspace{0.17em}=\hspace{0.17em}TC/HDL-C$

Atherogenic index $$\left(AI\right)\hspace{0.17em}=\hspace{0.17em}LDL-C/HDL-C$$

Hepatic indices were calculated using the following formulas:

Hepatic steatosis index $$\left(HSI\right)\hspace{0.17em}=\hspace{0.17em}8 \times (ALT/AST ratio)\hspace{0.17em}+\hspace{0.17em}BMI (+\hspace{0.17em}2, if female; + 2, if diabetes mellitus)$$.

$$ALD / NAFLD Index \left(ANI\right) = -58.5\hspace{0.17em}+\hspace{0.17em}0.637 \left(MCV\right)\hspace{0.17em}+\hspace{0.17em}3.91 (AST/ALT)-0.406 \left(BMI\right)\hspace{0.17em}+\hspace{0.17em}6.35$$

Aspartate aminotransferase-to-alanine aminotransferase ratio $\left(AAR\right)\hspace{0.17em}=\hspace{0.17em}AST/ ALT$

### Statistical analysis

Statistical analysis was conducted using IBM SPSS Statistics software (Version 24). The Kolmogorov-Smirnov test was used to assess the normality of the variables. Means and standard deviations (SD) were used to describe continuous variables, while numbers and percentages (%) were used to describe categorical variables. The t-test and the Mann-Whitney U test (for non-normal distributed variables) were used to compare continuous variables. The Chi-squared test was used to compare the categorical variables. Pearson’s correlation coefficient was used because of the observed linear correlation between mean anthropometric indices and hepatic and cardio-metabolic indices. Linear regression analysis was used in three models: Model 1: unadjusted; Model 2: adjusted for age and energy intake), and Model 3: adjusted for age, dietary intake (energy, fat, protein, and carbohydrate), and wrist circumference to determine the association between anthropometric indices and MAFLD. A *p*-value of less than 0.05 was considered statistically significant.

## Results

The study included 3190 males (40.7%) and 4646 females (59.2%). Among them, 212 males (33.0%) and 430 females (66.9%) were diagnosed with MAFLD. Table 1 presents a comparison of the population characteristics between individuals with and without MAFLD, categorized by gender (males and females). In both groups (MAFDL and non-MAFLD), the mean values for weight, height, wrist, systolic blood pressure, diastolic blood pressure, triglycerides (TG), alanine aminotransferase (ALT), aspartate aminotransferase (AST), gamma-glutamyl transferase (GGT), alkaline phosphatase (ALP), and hemoglobin were significantly higher in males than in females. The mean values of BMI, WC, HC, heart rate, and HDL were also significantly higher in females than in males.

**Table 1:** Characteristics of the study population (*n* = 7836)

Characteristics	Non-MAFLD (*n* = 7194)		*P* ^1^	MAFLD (*n* = 642)		*P* ^2^	*P* ^3^
	Male (*n* = 2978)	Female (*n* = 4216)		Male (*n* = 212)	Female (*n* = 430)		
**Age (years)**	46.0 (41.0–55.0)	45.0 (40.0–53.0)	< 0.001	46.0 (40.0–54.0)	48.0 (42.0–54.0)	0.16	< 0.001
**Gender %**	41.4%	58.6%		33.0%	67.0%		< 0.001
**Weight (cm)**	80.5 (71.0-99.5)	73.0 (64.0–84.0)	< 0.001	92.0(84.50-102.38)	83.0 (75.0–93.0)	< 0.001	< 0.001
**Height (cm)**	173.0 (169.0-177.5)	159.1 (155.3–163.0)	< 0.001	174.0 (169.35–178.0)	159.0(155.7-163.1)	< 0.001	< 0.001
**BMI (kg/m2)**	26.9 (23.9–30.0)	28.9 (25.4–32.8)	< 0.001	30.26(28.05–33.35)	32.9 (29.6–36.4)	< 0.001	< 0.001
**WC (cm)**	96.0 (88.0-103.0)	100.0 (91.5–108.0)	< 0.001	103.0 (98.0-111.0)	109.0 (102.0-115.1)	< 0.001	< 0.001
**Wrist (cm)**	18.0 (17.0-18.5)	17.0 (16.2–17.9)	< 0.001	18.50(17.70–19.0)	17.7 (16.9–18.5)	< 0.001	< 0.001
**HC (cm)**	100.0 (95.0-106.0)	105.5 (99.0-112.0)	< 0.001	105.90 (101.0-111.0)	111.0 (104.0-118.0)	< 0.001	< 0.001
**BP Systolic**	113.0 (105.0-121.0)	110.0 (100.0-120.0)	< 0.001	115.0 (106.0-123.0)	110.0(100.0-121.0)	< 0.001	0.017
**BP Diastolic**	72.0 (66.0–80.0)	70.0 (61.0–75.0)	< 0.001	75.0 (69.25-80.0)	70.0(65.0–78.0)	0.001	0.001
**Rate heart (n)**	75.0 (70.0–82.0)	78.0 (73.0–85.0)	< 0.001	76.50 (72.0–85.0)	79.0(74.0–86.0)	0.006	< 0.001
**TC (mg/dl)**	183.0 (161.0-209.0)	184.0 (162.0-211.0)	0.06	186.0 (161.5–207.0)	191.0(164.0-219.0)	0.05	0.07
**TG (mg/dl)**	147.0 (107.0-208.0)	121.0 (89.0-168.0)	< 0.001	173.50(130.0-244.0)	152.0(116.0-213.2)	0.001	< 0.001
**HDL (mg/dl)**	45.0 (39.0–52.0)	52.0 (45.0-60.75)	< 0.001	43.0 (37.0–49.0)	50.0(43.0–58.0)	< 0.001	0.001
**LDL (mg/dl)**	103.6 (83.0-124.6)	104.8 (86.0-126.0)	0.02	103.40(76.50-122.15)	104.4 (82.6-127.1)	0.11	0.09
**FBS (mg/dl)**	92.0 (87.0–99.0)	93.0 (86.0-100.0)	0.12	97.50(91.0-115.75)	104.0(92.0-128.2)	0.01	< 0.001
**ALT (U/L)**	22.0 (16.0–32.0)	14.0 (11.0–19.0)	< 0.001	31.0 (22.0–46.0)	17.0(13.0-23.2)	< 0.001	< 0.001
**AST (U/L)**	19.0 (16.0–23.0)	16.0 (13.0–19.0)	< 0.001	22.0 (16.0–28.0)	16.0(13.0–21.0)	< 0.001	0.054
**GGT (U/L)**	25.0 (19.0–36.0)	17.0 (14.0–24.0)	< 0.001	32.0 (26.0-49.75)	22.0(17.0–34.0)	< 0.001	< 0.001
**ALP (U/L)**	201.0 (174.0-235.2)	193.0 (160.0-234.0)	< 0.001	212.5(175.2-251.2)	209.0(167.0-245.5)	0.31	< 0.001
**Hemoglobin**	15.2 (14.4–15.9)	13.2 (12.4–13.9)	< 0.001	15.10(14.40–16.0)	13.2 (12.5–14.0)	< 0.001	0.017
**MCV (FL)**	85.3 (83.3–89.9)	84.9 (81.0-88.3)	< 0.001	85.30 (82.50-88.47)	83.8 (79.8–87.7)	< 0.001	0.001
**Energy (Kcal)**	3509.2 (2881.4-4184.1)	2770.79(2278.4-3361.8)	< 0.001	3421.4(28.62.0-3999.8)	2682.3 (2207.4-3247.9)	< 0.001	< 0.001

*BMI *Body mass index, *WC *Waist circumference, *HC *hip circumference, *BP *blood pressure, *TC *Total Cholesterol, *TG *Triglycerides, *HDL *High-density lipoprotein, *LDL *Low-density lipoprotein, *FBS *Fasting blood sugar, *ALT *Alanine aminotransferase, *AST *Aspartate transaminase, *GGT *Gamma-glutamyl transferase, *ALP *Alkaline phosphatase

P^1^: *P*-value between male and female in Non-MAFLD

P^2^: *P*-value between male and female in MAFLD

P^3^: *P*-value between Non-MAFLD and NAFLD

T-test (variables with normal distribution) and the Mann-Whitney U test (variables with non-normal distribution) were used to compare continuous variables

Chi-squared test was used to compare the categorical variables

Patients with MAFLD showed significant differences compared to non-MAFLD individuals, particularly in anthropometric indices such as weight, height, BMI, wrist, WC and HC in both sexes (Table 1). Moreover, in all patients with MAFLD, age, systolic blood pressure (SBP), diastolic blood pressure (DBP), heart rate, triglycerides (TG), fasting blood sugar (FBS), alanine aminotransferase (ALT), gamma-glutamyl transferase (GGT), alkaline phosphatase (ALP), were significantly higher compared to those without MAFLD. HDL levels were significantly lower in patients with MAFLD, whereas total cholesterol (TC), low-density lipoprotein (LDL), and aspartate transaminase (AST) levels showed no significant differences between the two groups.

Pearson’s correlation coefficients of anthropometric indices with hepatic and cardio-metabolic indices in MAFLD patients are summarized in Table 2. Anthropometric indices demonstrated statistically significant correlations with several variables, except for the pairs “CMI/RFM”, “TyG index/RFM”, “TIMI risk index/VAI”, and “AI/WWI”. Among the observed correlations, the strongest positive ones were between WC/BMI/BAI/BRI/AVI and HIS/TyG-BMI/TyG-WC, as well as VAI and AIP/LAP/CMI/TyG Index.On the other hand, WHtR exhibited the strongest negative correlation with HIS, TyG-BMI, and TyG-WC.

**Table 2:** Pearson’s correlation coefficients between anthropometric indices and hepatic and cardio-metabolic indices among males and females

Variables		WC	BMI	WHR	WHtR	BAI	VAI	WWI	CI	BRI	RFM	AVI
**FBS**	**r**	0.279	0.140	0.177	-0.173	0.166	0.158	0.149	0.166	0.176	0.115	0.180
	**p**	< 0.001	< 0.001	< 0.001	< 0.001	< 0.001	< 0.001	< 0.001	< 0.001	< 0.001	< 0.001	< 0.001
**MAP**	**r**	0.162	0.136	0.099	-0.103	0.068	0.076	0.011	0.066	0.095	-0.054	0.158
	**p**	< 0.001	< 0.001	< 0.001	< 0.001	< 0.001	< 0.001	0.337	< 0.001	< 0.001	< 0.001	< 0.001
**AAR**	**r**	-0.212	-0.191	-0.097	0.113	-0.045	-0.124	0.075	-0.017	-0.088	0.117	-0.200
	**p**	< 0.001	< 0.001	< 0.001	< 0.001	< 0.001	< 0.001	< 0.001	< 0.001	< 0.001	< 0.001	< 0.001
**ANI**	**r**	-0.479	-0.533	-0.602	0.598	-0.623	-0.100	-0.442	-0.348	-0.595	-0.683	-0.478
	**p**	< 0.001	< 0.001	< 0.001	< 0.001	< 0.001	< 0.001	< 0.001	< 0.001	< 0.001	< 0.001	< 0.001
**HIS**	**r**	0.817	0.903	0.777	-0770	0.722	0.186	0.284	0306	0.770	0.500	0.813
	**P**	< 0.001	< 0.001	< 0.001	< 0.001	< 0.001	< 0.001	< 0.001	< 0.001	< 0.001	< 0.001	< 0.001
**AIP**	**r**	0.221	0.171	0.106	-0.126	0.054	0.813	-0.027	0.073	0.095	-0.102	0.205
	**p**	< 0.001	< 0.001	< 0.001	< 0.001	< 0.001	< 0.001	< 0.001	< 0.001	< 0.001	< 0.001	< 0.001
**LAP**	**r**	0.533	0.458	0.472	-0.474	0.423	0.861	0.287	0.359	0.464	0.276	0.527
	**p**	< 0.001	< 0.001	< 0.001	< 0.001	< 0.001	< 0.001	< 0.001	< 0.001	< 0.001	< 0.001	< 0.001
**CMI**	**r**	0.251	0.210	0.177	-0.188	0.138	0.965	0.058	0.125	0.169	0.007	0.242
	**p**	< 0.001	< 0.001	< 0.001	< 0.001	< 0.001	< 0.001	< 0.001	< 0.001	< 0.001	0.534	< 0.001
**LCI**	**r**	0.133	0.096	0.086	-0.100	0.063	0.499	0.034	0.079	0.078	-0.030	0.122
	**p**	< 0.001	< 0.001	< 0.001	< 0.001	< 0.001	< 0.001	0.003	< 0.001	< 0.001	0.007	< 0.001
**TyG Index**	**r**	0.271	0.216	0.190	-0.208	0.148	0.766	0.074	0.151	0.179	0.005	0.256
	**p**	< 0.001	< 0.001	< 0.001	< 0.001	< 0.001	< 0.001	< 0.001	< 0.001	< 0.001	0.637	< 0.001
**TyG-BMI**	**r**	0.866	0.955	0.823	-0.808	0.765	0.365	0.304	0.330	0.820	0.507	0.865
	**p**	< 0.001	< 0.001	< 0.001	< 0.001	< 0.001	< 0.001	< 0.001	< 0.001	< 0.001	< 0.001	< 0.001
**TyG-WC**	**r**	0.916	0.799	0.813	-0.808	0.730	0.491	0.481	0.602	0.804	0.464	0.908
	**p**	< 0.001	< 0.001	< 0.001	< 0.001	< 0.001	< 0.001	< 0.001	< 0.001	< 0.001	< 0.001	< 0.001
**TIMI risk index**	**r**	0.034	-0.118	0.102	-0.091	0.125	-0.014	0.349	0.341	0.105	0.095	0.036
	**p**	0.003	< 0.001	< 0.001	< 0.001	< 0.001	0.229	< 0.001	< 0.001	< 0.001	< 0.001	0.001
**CRI**	**r**	0.161	0.115	0.066	-0.084	0.024	0.669	-0.029	0.054	0.056	-0.116	0.147
	**p**	< 0.001	< 0.001	< 0.001	< 0.001	0.034	< 0.001	0.011	< 0.001	< 0.001	< 0.001	< 0.001
**AI**	**r**	0.112	0.076	0.056	-0.067	0.030	0.123	0.003	0.056	0.049	-0.069	0.101
	**p**	< 0.001	< 0.001	< 0.001	< 0.001	0.007	< 0.001	0.777	< 0.001	< 0.001	< 0.001	< 0.001

*FBS *Fasting blood pressure, *MAP *Mean Arterial Pressure, ANI; ALD/NAFLD Index, *HIS *Hepatic steatosis index, *AIP *Atherogenic index of plasma, LAP; *CMI *Cardiometabolic index, *LCI *The lipoprotein combine index, *AI *Atherogenic index, *TyG *The triglyceride-glucose

The logistic regression analysis revealed that all anthropometric indices were significantly associated with MAFLD (*P* < 0.001) even after ajusting for potential confounding variables. However, WWI had the strongest association with MAFLD in males [ORs 1.747 (95% CI, 1.402–1.178)] and females [ORs 1.743 (95% CI, 1.506–2.018)]. After adjusting for potential confounding variables such as age and energy intake in Model 2, and also for age, dietary intake (energy, fat, protein, and carbohydrate), and wrist circumference in Model 3, the OR (95% CI) for WWI demonstrated a significant increase (*P* < 0.001) (Table 3).

**Table 3:** Logistic regression analyses for investigation of the association between anthropometric indices and MAFLD

Variables	Model 1			Model 2			Model 3		
	Odd ratio	(95% CI)	*P*	Odd ratio	(95% CI)	*P*	Odd ratio	(95% CI)	*P*
**Male**									
** WC**	1.067	(1.055–1.080)	< 0.001	1.070	(1.057–1.083)	< 0.001	1.067	(1.050–1.085)	< 0.001
** BMI**	1.178	(1.144–1.213)	< 0.001	1.182	(1.148–1.217)	< 0.001	1.184	(1.135–1.235)	< 0.001
** WHR**	1.110	(1.083–1.138)	< 0.001	1.139	(1.108–1.170)	< 0.001	1.114	(1.081–1.147)	< 0.001
** WHtR**	0.019	(0.009–0.041)	< 0.001	0.014	(0.006–0.032)	< 0.001	0.021	(0.008–0.057)	< 0.001
** BAI**	1.133	(1.104–1.162)	< 0.001	1.146	(1.116–1.176)	< 0.001	1.117	(1.082–1.153)	< 0.001
** VAI**	1.082	(1.040–1.127)	< 0.001	1.083	(1.040–1.129)	< 0.001	1.065	(1.020–1.111)	0.004
** WWI**	1.747	(1.402–1.178)	< 0.001	2.178	(1.697–2.796)	< 0.001	1.818	(1.399–2.364)	< 0.001
** CI**	1.061	(1.040–1.082)	< 0.001	1.079	(1.056–1.103)	< 0.001	1.057	(1.033–1.082)	< 0.001
** BRI**	1.558	(1.428-1.700)	< 0.001	1.607	(1.470–1.758)	< 0.001	1.491	(1.332–1.668)	< 0.001
** RFM**	1.220	(1.173–1.268)	< 0.001	1.236	(1.188–1.287)	< 0.001	1.212	(1.154–1.273)	< 0.001
** AVI**	1.161	(1.129–1.193)	< 0.001	1.168	(1.136–1.201)	< 0.001	1.153	(1.110–1.198)	< 0.001
**Female**									
** WC**	1.071	(1.061–1.080)	< 0.001	1.070	(1.061–1.080)	< 0.001	1.060	(1.048–1.072)	< 0.001
** BMI**	1.143	(1.122–1.165)	< 0.001	1.148	(1.127–1.169)	< 0.001	1.126	(1.099–1.154)	< 0.001
** WHR**	1.090	(1.074–1.107)	< 0.001	1.093	(1.076–1.110)	< 0.001	1.083	(1.065–1.101)	< 0.001
** WHtR**	0.008	(0.004–0.015)	< 0.001	0.008	(0.004–0.016)	< 0.001	0.018	(0.008–0.038)	< 0.001
** BAI**	1.128	(1.109–1.147)	< 0.001	1.127	(1.108–1.146)	< 0.001	1.099	(1.079–1.121)	< 0.001
** VAI**	1.176	(1.132–1.223)	< 0.001	1.173	(1.128–1.220)	< 0.001	1.150	(1.105–1.196)	< 0.001
** WWI**	1.743	(1.506–2.018)	< 0.001	1.748	(1.486–2.057)	< 0.001	1.760	(1.486–2.083)	< 0.001
** CI**	1.058	(1.044–1.072)	< 0.001	1.059	(1.043–1.074)	< 0.001	1.052	(1.036–1.068)	< 0.001
** BRI**	1.488	(1.411–1.568)	< 0.001	1.483	(1.407–1.564)	< 0.001	1.377	(1.294–1.466)	< 0.001
** RFM**	1.274	(1.232–1.317)	< 0.001	1.272	(1.230–1.315)	< 0.001	1.223	(1.177–1.270)	< 0.001
** AVI**	1.168	(1.145–1.192)	< 0.001	1.167	(1.144–1.191)	< 0.001	1.140	(1.112–1.169)	< 0.001

Model 1: unadjusted; Model 2: adjusted for age and energy intake; Model 3: adjusted for age, dietary intake (energy, fat, protein and carbohydrate) and wrist circumference. *WC* Waist circumference, *BMI *Body mass index, *WHR *Waist-to-hip ratio, *WHtR *Waist-to-height ratio, *BAI *Body adiposity index, *VAI *Visceral adiposity index, *WWI *Weight-adjusted Waist Index, *CI *Conicity index, *BRI *Body Roundness Index, *RFM *Relative fat mass, *AVI *Abdominal volume index

Table 4 displays the adjusted ORs of MAFLD risk conditions with one SD increase in anthropometric indices. Odds ratios were adjusted for age, dietary intake (energy, fat, protein and carbohydrate) and wrist circumference. All anthropometric indices were significantly associated with MAFLD risk factors except VAI (OR = 1.02, 95% CI: 0.97–1.07; *p* = 0.472) and CI (OR = 1.02, 95% CI: 1.00-1.04; *p* = 0.069) for male hypertension and CI (OR = 1.01, 95% CI: 1.00-1.03; *p* = 0.232) for female hypertension. Also, our analysis identified VAI as a powerful predictor for MAFLD risk factors, demonstrating a particularly strong association with the presence of at least one risk factor in both males (OR = 11.79, 95% CI: 9.57–14.51) and females (OR = 6.57, 95% CI: 5.87–7.76).Similarly, WWI showed a significant correlation with MAFLD risk factors, notably diabetes in both males(OR = 3.81, 95% CI: 2.40–6.04) and females (OR = 2.28, 95% CI: 1.73–3.01). Conversely, WHtR demonstrated a notably weaker association with MAFLD risk factors, particularly diabetes in both males(OR = 0.007, 95% CI: 0.01–0.051) and females (OR = 0.02, 95% CI: 0.01–0.08).

**Table 4:** Odd ratios and 95% CI for MAFLD risk conditions corresponding to one SD increase in anthropometric indices

Variables	Diabetes			Hypertension			Dyslipidemia			≥ 1 risk factor			≥ 2 risk factor		
	OR	(95% CI)	*P*	OR	(95% CI)	*P*	OR	(95% CI)	*P*	OR	(95% CI)	*P*	OR	(95% CI)	*P*
Male															
** WC**	1.08	(1.05–1.11)	< 0.001	1.03	(1.01–1.04)	< 0.001	1.04	(1.03–1.05)	< 0.001	1.04	(1.03–1.05)	< 0.001	1.05	(1.03–1.06)	< 0.001
** BMI**	1.14	(1.06–1.23)	< 0.001	1.10	(1.06–1.14)	< 0.001	1.09	(1.06–1.12)	< 0.001	1.10	(1.07–1.13)	< 0.001	1.14	(1.10–1.19)	< 0.001
** WHR**	1.20	(1.14–1.27)	< 0.001	1.04	(1.02–1.06)	< 0.001	1.07	(1.06–1.09)	< 0.001	1.08	(1.06–1.10)	< 0.001	1.08	(1.05–1.10)	< 0.001
** WHtR**	0.007	(0.01–0.05)	< 0.001	0.17	(0.08–0.35)	< 0.001	0.12	(0.08–0.19)	< 0.001	0.11	(0.07–0.17)	< 0.001	0.06	(0.03–0.14)	< 0.001
** BAI**	1.15	(1.09–1.21)	< 0.001	1.05	(1.03–1.09)	< 0.001	1.07	(1.05–1.10)	< 0.001	1.08	(1.06–1.10)	< 0.001	1.09	(1.06–1.12)	< 0.001
** VAI**	1.13	(1.07–1.19)	< 0.001	1.02	(0.97–1.07)	0.472	14.35	(11.57–17.79)	< 0.001	11.79	(9.57–14.51)	< 0.001	1.10	(1.06–1.15)	< 0.001
** WWI**	3.81	(2.40–6.04)	< 0.001	1.27	(1.03–1.56)	0.024	1.61	(1.40–1.86)	< 0.001	1.69	(1.46–1.96)	< 0.001	1.59	(1.28–1.98)	< 0.001
** CI**	1.13	(1.08–1.18)	< 0.001	1.02	(1.00-1.04)	0.069	1.04	(1.03–1.05)	< 0.001	1.05	(1.03–1.06)	< 0.001	1.04	(1.02–1.06)	< 0.001
** BRI**	1.61	(1.34–1.93)	< 0.001	1.21	(1.10–1.33)	< 0.001	1.30	(1.21–1.40)	< 0.001	1.33	(1.23–1.44)	< 0.001	1.37	(1.24–1.51)	< 0.001
** RFM**	1.28	(1.17–1.40)	< 0.001	1.09	(1.05–1.13)	< 0.001	1.11	(1.09–1.14)	< 0.001	1.12	(1.09–1.14)	< 0.001	1.15	(1.10–1.19)	< 0.001
** AVI**	1.19	(1.11–1.26)	< 0.001	1.06	(1.03–1.10)	< 0.001	1.09	(1.07–1.12)	< 0.001	1.10	(1.07–1.13)	< 0.001	1.11	(1.07–1.15)	< 0.001
**Female**															
** WC**	1.06	(1.04–1.08)	< 0.001	1.060	(1.05–1.07)	< 0.001	1.03	(1.02–1.04)	< 0.001	1.03	(1.02–1.04)	< 0.001	1.04	(1.03–1.05)	< 0.001
** BMI**	1.08	(1.04–1.12)	< 0.001	1.13	(1.10–1.15)	< 0.001	1.05	(1.03–1.07)	< 0.001	1.05	(1.04–1.07)	< 0.001	1.07	(1.04–1.10)	< 0.001
** WHR**	1.12	(1.09–1.15)	< 0.001	1.02	(1.01–1.04)	0.005	1.06	(1.05–1.07)	< 0.001	1.06	(1.05–1.07)	< 0.001	1.07	(1.05–1.09)	< 0.001
** WHtR**	0.02	(0.01–0.08)	< 0.001	0.02	(0.01–0.04)	< 0.001	0.15	(0.10–0.23)	< 0.001	0.15	(0.10–0.22)	< 0.001	0.10	(0.05–0.20)	< 0.001
** BAI**	1.08	(1.05–1.11)	< 0.001	1.10	(1.08–1.12)	< 0.001	1.05	(1.04–1.06)	< 0.001	1.05	(1.04–1.07)	< 0.001	1.06	(1.04–1.08)	< 0.001
** VAI**	1.20	(1.14–1.26)	< 0.001	1.15	(1.10–1.12)	< 0.001	7.41	(6.44–8.52)	< 0.001	6.75	(5.87–7.76)	< 0.001	1.20	(1.15–1.25)	< 0.001
** WWI**	2.28	(1.73–3.01)	< 0.001	1.76	(1.49–2.08)	< 0.001	1.51	(1.35–1.68)	< 0.001	1.52	(1.36–1.70)	< 0.001	1.60	(1.34–1.91)	< 0.001
** CI**	1.09	(1.06–1.11)	< 0.001	1.01	(1.00-1.03)	0.232	1.04	(1.03–1.05)	< 0.001	1.04	(1.03–1.05)	< 0.001	1.04	(1.03–1.06)	< 0.001
** BRI**	1.32	(1.20–1.45)	< 0.001	1.38	(1.29–1.47)	< 0.001	1.18	(1.13–1.23)	< 0.001	1.20	(1.14–1.26)	< 0.001	1.22	(1.15–1.31)	< 0.001
** RFM**	1.20	(1.13–1.28)	< 0.001	1.22	(1.18–1.27)	< 0.001	1.10	(1.08–1.12)	< 0.001	1.10	(1.08–1.12)	< 0.001	1.12	(1.08–1.17)	< 0.001
** AVI**	1.13	(1.09–1.18)	< 0.001	1.14	(1.11–1.17)	< 0.001	1.07	(1.05–1.09)	< 0.001	1.08	(1.06–1.10)	< 0.001	1.09	(1.06–1.2)	< 0.001

*WC *Waist circumference, *BMI *Body mass index, *WHR *Waist-to-hip ratio, *WHtR *Waist-to-height ratio, *BAI *Body adiposity index, *VAI *Visceral adiposity index, *WWI *Weight-adjusted Waist Index, *CI *Conicity index, *BRI *Body Roundness Index, *RFM *Relative fat mass. Odds Ratio Adjusted for age, dietary intake (energy, fat, protein and carbohydrate) and wrist circumference

**Fig. 2:**
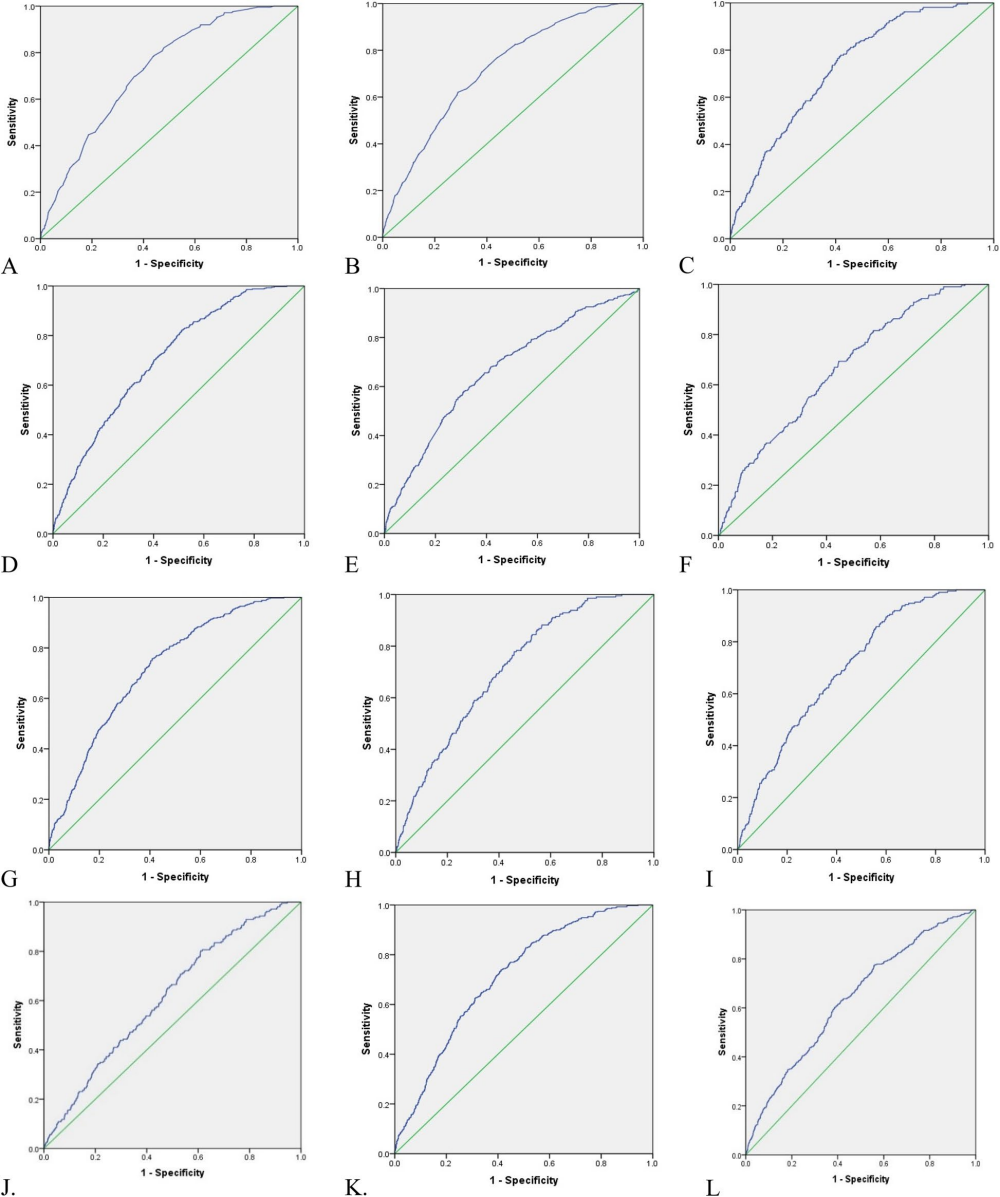
The area under the curve (AUC) for (**A**) WC in male, (**B**) WC in female, (**C**) BMI in male, (**D**) BMI in female, (**E**) WHR in male, (**F**) WHR in female, (**G**) WHtR in male, (**H**) WHtR in female, **(I)** BAI in male, **(J)** BAI in female, **K.** VAI in male, **L.** VAI in female

**Fig. 3:**
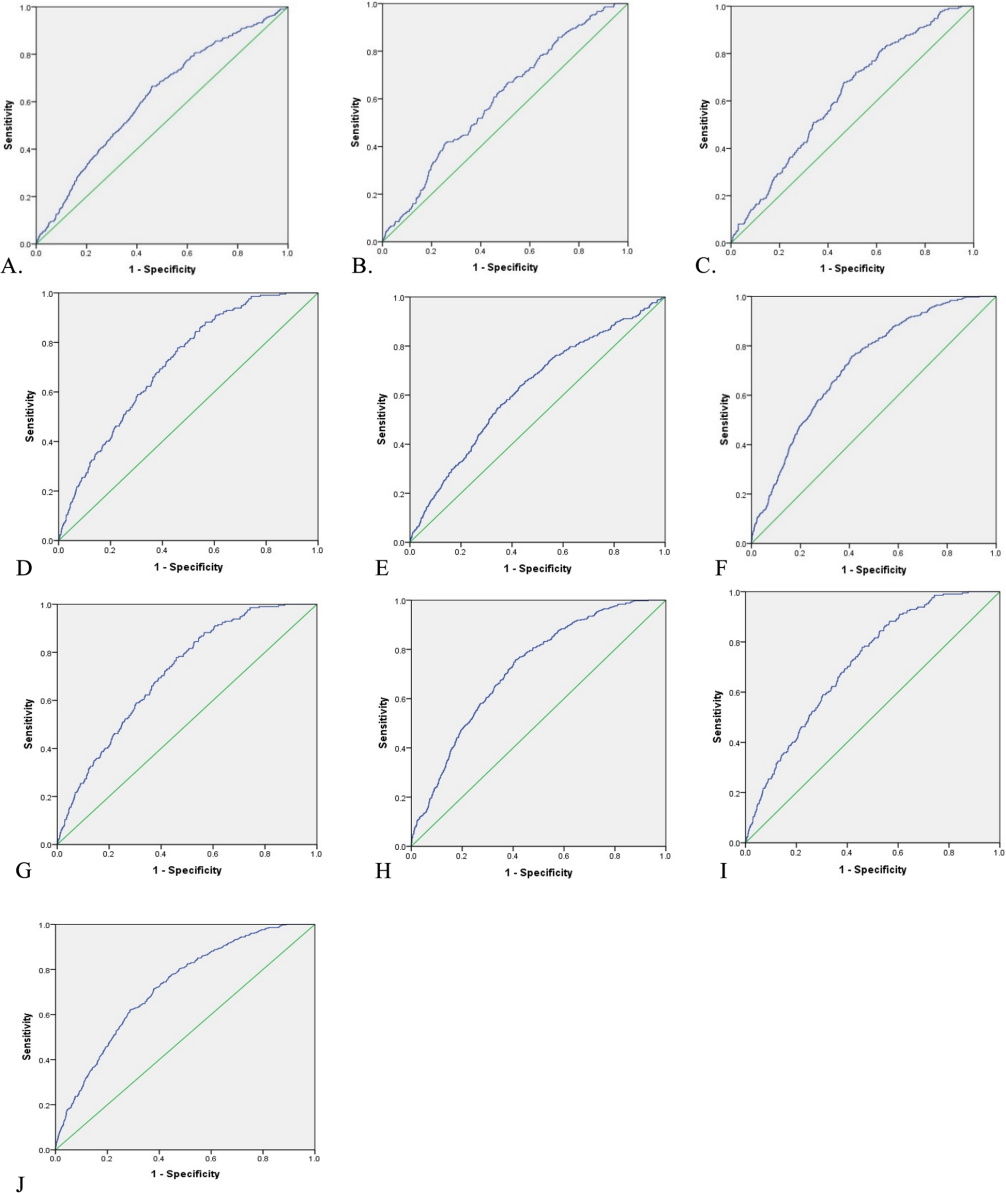
The area under the curve (AUC) for (**A**) WWI in male, (**B**) WWI in female, (**C**) CI in male, (**D**) CI in female, (**E**) BRI in male, (**F**) BRI in female, (**G**) RFM in male, (**H**) RFM in female, (**I**) AVI in male, (**J**) AVI in female

Estimated gender-specific optimal cut-off values for predicting MAFLD risk were used as binary classifiers for establishing the new cut-off points for the anthropometric indicators. The optimal cut-off values of anthropometric indices for predicting MAFLD risk were determined for WC (102.25 cm for males and 101.45 cm for females), BMI (27.80 kg/m^2^ for males and 28.75 kg/m^2^ for females), WHR (0.96 for both males and females), WHtR (0.56 for males and 0.63 for females), BAI (23.24 for males and 32.97 for females), VAI (1.64 for males and 1.88 for females), WWI (10.63 for males and 11.71 for females), CI (1.29 for males and 1.36 for females), BRI (4.52 for males and 6.45 for females), RFM (28.18 for males and 44.91 for females) and AVI (18.85 for males and for 21.37 females). VAI in males (sen:77%, spe:60%, YI; 0.37) and RFM in female (sen:76%, spe:59%, YI; 0.35) had the highest sensitivity and specificity than other anthropometric indices (Table 5). The ROC curve for the investigated indicators shown in Figs. 2 and 3.

**Table 5:** Optimal cut-off value for anthropometric indices predictive of MAFLD

Variables	MAFLD									
	Male					Female				
	Cutoff	AUC	Sen (%)	Spe (%)	YI	Cutoff	AUC	Sen (%)	Spe (%)	YI
**WC**	102.25	0.725	67	61	0.28	101.45	0.722	77	56	0.33
**BMI**	27.80	0.730	78	58	0.36	28.75	0.709	82	49	0.31
**WHR**	0.96	0.668	69	53	0.22	0.96	0.666	66	61	0.27
**WHtR**	0.56	0.713	78	54	0.32	0.63	0.720	79	54	0.33
**BAI**	23.24	0.700	87	44	0.30	32.97	0.712	74	59	0.33
**VAI**	1.64	0.615	77	60	0.37	1.88	0.638	78	44	0.22
**WWI**	10.63	0.600	67	49	0.16	11.71	0.616	67	54	0.21
**CI**	1.29	0.620	68	53	0.21	1.36	0.624	64	57	0.21
**BRI**	4.52	0.713	78	54	0.22	6.45	0.720	76	59	0.25
**RFM**	28.18	0.713	78	54	0.32	44.91	0.720	76	59	0.35
**AVI**	18.85	0.726	80	55	0.35	21.37	0.722	72	62	0.34

*AUC *Area under the ROC Curve, *WC *Waist circumference, *BMI *Body mass index, *WHR *Waist-to-hip ratio, *WHtR *Waist-to-height ratio, *BAI *Body adiposity index, *VAI *Visceral adiposity index, *WWI *Weight-adjusted Waist Index, *CI *Conicity index, *BRI* Body Roundness Index, *RFM *Relative fat mass

## Discussion

This cross-sectional study demonstrated that anthropometric indices are significant in predicting Metabolic Associated Fatty Liver Disease (MAFLD).The precise causes and factors contributing to the development and severity of MAFLD are still not well understood. Given the high prevalence rate of MAFLD worldwide, early diagnosis and intervention are essential to alleviate the burden of this disease. The occurrence and progression of fatty liver are significantly influenced by metabolic disorders, nutrition, and lifestyle [20]. The presence of cardiovascular disease along with certain anthropometric indices like body mass index (BMI), waist circumference (WC), and waist-to-height ratio (WHtR) may be effective in predicting MAFLD risk [11].

In this cross-sectional study, various anthropometric indices were measured, including WC, BMI, WHR, WHtR, BAI, VAI, WWI, CI, BRI, RFM, and AVI. In our study, logistic regression analysis assessed the association between these indices and MAFLD. The results revealed that WWI had the highest odds ratios in males and females, both before and after adjusting for confounding factors. In 2018, the weight-adjusted waist index (WWI) was initially proposed as a measurement of central obesity that considers both fat and muscle mass components, independent of the body mass index [21]. Multiple studies have demonstrated a notable correlation between WWI and various health conditions such as cardiovascular disease, chronic kidney disease, hepatic steatosis, and hepatic fibrosis [21, 22].

Our study revealed a strong positive correlation between WC, BMI, BAI, BRI and AVI with HIS, TyG-BMI and TyG-WC, as well as between VAI with AIP, LAP, CMI and TyG Index, as indicated by Pearson’s correlation coefficients. However, some studies have shown that LAP is the best predictor of obese individuals with MAFLD, with the maximum ORs value [11, 23]. Also, In a cross-sectional study that included 7968 participants, Yang et al. [24] discovered that TyG-BMI, BMI, TyG, TG/HDL-C, and TG are five significant predictors of the risk of MAFLD.

The highest odds ratios were found for VAI in relation to dyslipidemia and having one or more risk factors for MAFLD. In addition, the cut-off values for anthropometric indices used to predict MAFLD, varied by gender. According to our results, VAI in males and RFM in females had the highest sensitivity and specificity in predicting MAFLD risk, with the cut-off values of 1.64 and 44.91, respectively. The visceral adiposity index (VAI) has been suggested as a reliable indicator for the accumulation and dysfunction of visceral fat, and it has been demonstrated to have a strong correlation with the presence of type 2 diabetes, cardiovascular events, and hepatic fibrosis [25]. Recently, Ismaiel et al. [26] in a meta-analysis study, demonstrated that the VAI has a predictive value for diagnosing NAFLD and NASH, with noticeably higher values observed in adult patients with NAFLD.

The authors of a study suggested that a higher risk of developing NAFLD over a 4-year period is associated with larger areas of visceral adipose tissue, and that the distribution of fat has a greater impact on NAFLD than the content of the fat. This mechanism explains the relationship between VAI and NAFLD [26]. Additionally, the relative fat mass (RFM) is a basic linear equation that relies on the ratio of height to waist circumference [27]. One study has demonstrated that obesity defined by RFM is more effective in predicting dyslipidemia, hypertension, and abnormal secretion of adipokines. However, it does not offer additional benefits in predicting non-alcoholic fatty liver disease (NAFLD) or liver damage compared to obesity defined by BMI [28].

For both sexes, the optimal cut-off values for BMI were lower (27.80 kg/m^2^ in males, 28.75 kg/m^2^ in female) than the values indicating obesity (> 30 kg/m^2^). The BMI cutoff points obtained by Jing Liu et al. [23], Cai et al. [11], and Yang et al. [24] were 25.39, 24, and 24.61 kg/m2 respectively, which were lower than our findings. It is well accepted that BMI is closely related to the risk of fatty liver disease and is an important factor in determining negative clinical outcomes [10]. On the contrary, Jing Liu et al. [23] suggested that BMI is not a valuable diagnostic indicator for MAFLD. Nevertheless, the correlation between MAFLD and BMI is intricate and can be affected by numerous factors, including one’s racial or ethnic background and variations in specific genes [10].

Furthermore, in the present study, the optimal cut-off value for WC, which is an indicator of the degree of visceral fat accumulation, in screening for MAFLD was 102.25 cm (sensitivity 67%, specificity 61%) in males and 101.45 cm (sensitivity 77%, specificity 56%) in females, which was higher than common threshold values for obesity. In other Asian countries, WC and WHtR cutoff values for predicting NAFLD were lower than our results [29–31].Visceral fat has a strong correlation with fatty liver, and this condition is linked to a high incidence of lifestyle-related diseases such as hypertension, hyperlipidemia, and diabetes [29].

To the best of our knowledge, this is the first study to evaluate the appropriate cutoff points for anthropometric indices to predict MAFLD risk in the Iranian population. The strength of this study is the inclusion of a large number of individuals who have been diagnosed with MAFLD. In addition, we used updated and novel anthropometric indices for the evaluation of optimal cut-off values. There are several limitations of this study that need to be mentioned. First, due to the cross-sectional study design, we were unable to establish any causal relationships. Second, it is important to note that our findings may not be applicable to other ethnicities, thus necessitating further research on diverse ethnic groups.

## Conclusion

In conclusion, we found that all eleven anthropometric indices have diagnostic significance for MAFLD. Principally, WWI was found to be the strongest indicator for predicting MAFLD risk among Iranian adults. Additionally, VAI and RFM emerged as significant indicators, particularly when considering their sensitivity, specificity, and Youden’s Index. Few studies have examined the cutoff points of novel anthropometric indicators for predicting MAFLD risk. Identifying specific indicators with cutoff values for different ethnic and racial groups could aid in the early diagnosis of MAFLD and be helpful in preventing or managing its progression.

## Data Availability

All data generated or analysed during this study are included in this published article.

## Abbreviations

MAFLD: Metabolic associated fatty liver disease
NAFLD: Non-alcoholic fatty liver disease
ALD: Alcoholic liver disease
NASH: Non-alcoholic steatohepatitis
BMI: Body mass index
WC: Waist circumference
HC: Hip circumference
WHR: Waist-to-hip ratio
WHtR: Waist-to-height ratio
BAI: Body adiposity index
VAI: Visceral adiposity index
WWI: Weight-adjusted waist index
CI: Conicity index
BRI: Body roundness index
RFM: Relative fat mass
AVI: Abdominal volume index
CBC: Complete blood count
SBP: Systolic blood pressure
DBP: Diastolic blood pressure
FBG: Fasting blood glucose
TC: Total cholesterol
TG: Triglyceride
LDL-C: Low-density lipoprotein cholesterol
HDL-C: High-density lipoprotein cholesterol
MAP: Mean Arterial Pressure
ANI: ALD/NAFLD Index
HIS: Hepatic steatosis index
AIP: Atherogenic index of plasma
LAP: Lipid accumulation product
CMI: Cardiometabolic index
LCI: Lipoprotein combine index
AI: Atherogenic index
TyG: Triglyceride and glucose index
FPG: Fasting plasma glucose
ALT: Alanine aminotransferase
AST: Aspartate aminotransferase
GGT: Gamma-glutamyl transpeptidase
